# Genomic Selection in Rubber Tree Breeding: A Comparison of Models and Methods for Managing G×E Interactions

**DOI:** 10.3389/fpls.2019.01353

**Published:** 2019-10-25

**Authors:** Livia M. Souza, Felipe R. Francisco, Paulo S. Gonçalves, Erivaldo J. Scaloppi Junior, Vincent Le Guen, Roberto Fritsche-Neto, Anete P. Souza

**Affiliations:** ^1^Molecular Biology and Genetic Engineering Center (CBMEG), University of Campinas (UNICAMP), Campinas, Brazil; ^2^Center of Rubber Tree and Agroforestry Systems, Agronomic Institute (IAC), Votuporanga, Brazil; ^3^Centre de Coopération Internationale en Recherche Agronomique pour le Développement (CIRAD), UMR AGAP, Montpellier, France; ^4^Departamento de Genética, Escola Superior de Agricultura “Luiz de Queiroz” Universidade de São Paulo (ESALQ/USP), Piracicaba, Brazil; ^5^Department of Plant Biology, Biology Institute, University of Campinas (UNICAMP), Campinas, Brazil

**Keywords:** *Hevea brasiliensis*, breeding, multienvironment, single nucleotide, genotyping

## Abstract

Several genomic prediction models combining genotype × environment (G×E) interactions have recently been developed and used for genomic selection (GS) in plant breeding programs. G×E interactions reduce selection accuracy and limit genetic gains in plant breeding. Two data sets were used to compare the prediction abilities of multienvironment G×E genomic models and two kernel methods. Specifically, a linear kernel, or GB (genomic best linear unbiased predictor [GBLUP]), and a nonlinear kernel, or Gaussian kernel (GK), were used to compare the prediction accuracies (PAs) of four genomic prediction models: 1) a single-environment, main genotypic effect model (SM); 2) a multienvironment, main genotypic effect model (MM); 3) a multienvironment, single-variance G×E deviation model (MDs); and 4) a multienvironment, environment-specific variance G×E deviation model (MDe). We evaluated the utility of genomic selection (GS) for 435 individual rubber trees at two sites and genotyped the individuals *via* genotyping-by-sequencing (GBS) of single-nucleotide polymorphisms (SNPs). Prediction models were used to estimate stem circumference (SC) during the first 4 years of tree development in conjunction with a broad-sense heritability (*H* ^2^) of 0.60. Applying the model (SM, MM, MDs, and MDe) and kernel method (GB and GK) combinations to the rubber tree data revealed that the multienvironment models were superior to the single-environment genomic models, regardless of the kernel (GB or GK) used, suggesting that introducing interactions between markers and environmental conditions increases the proportion of variance explained by the model and, more importantly, the PA. Compared with the classic breeding method (CBM), methods in which GS is incorporated resulted in a 5-fold increase in response to selection for SC with multienvironment GS (MM, MDe, or MDs). Furthermore, GS resulted in a more balanced selection response for SC and contributed to a reduction in selection time when used in conjunction with traditional genetic breeding programs. Given the rapid advances in genotyping methods and their declining costs and given the overall costs of large-scale progeny testing and shortened breeding cycles, we expect GS to be implemented in rubber tree breeding programs.

## Introduction

Rubber tree (*Hevea brasiliensis*) breeding programs are generally characterized by breeding cycles of 25–30 years and include the crosses, evaluation, and selection of field progeny as well as the propagation of selected superior materials (Gonçalves et al., 2006). Compared with animal and annual crop species breeding, forest tree breeding is still in its infancy; the most advanced programs are in their third or fourth cycle of breeding, with very little differentiation occurring between the bred populations and natural populations (Isik, 2014). Rubber tree breeding programs are complex and costly because the large size of these trees necessitates experiments over large tracts of land, and progeny tests are expensive to establish, manage over many years, and evaluate *via* measurements.

The main objective of rubber tree breeding is the development of early selection methods that support the accurate prediction of mature phenotypes at a young stage; these methods are therefore important for shortening breeding cycles and thus improving the cost efficiency of such breeding programs. The time taken to derive a *Hevea* through breeding must be substantially reduced. Priyadarshan (2017) proposed two strategies: 1) truncating the breeding steps that follow conventional means and 2) incorporating genomics into breeding programs specifically to identify high-yielding genotypes in half-sib, full-sib, and polycross seedlings during the juvenile stage to minimize both space and time.

Classic plant breeding programs depend principally on phenotypic evaluation in several environments; selection and recombination are based on the resulting data and genotype information when available. Genomic selection (GS), a new approach in which whole-genome molecular markers are used, has the potential to quickly improve complex traits with low heritability, significantly reduce the cost of line and hybrid development and increase yields in reduced amounts of time, allowing improvements to quantitative traits within large plant breeding populations (Meuwissen et al., 2001).

Genomic prediction combines phenotypic and pedigree data with marker data in efforts to increase the prediction accuracy (PA) for breeding and genotypic values. This method depends on dense genome-wide marker coverage to produce genomic estimated breeding values (GEBVs) from a comprehensive analysis of all available markers.

According to Lorenz et al. (2011), the accuracy of GS, which is measured as the correlation between GEBVs and true breeding values, is affected by the relationship between the training (TRN) and testing (TST) sets, the number of individuals in the TRN set, linkage disequilibrium (LD) between the markers and quantitative trait loci (QTLs), the distribution of the underlying QTL effects, the statistical method used to estimate the GEBVs, and the trait heritability.

According to Meuwissen et al. (2001), GS has received increasing interest from forest tree breeders. In reports of initial experiments involving *Pinus* and *Eucalyptus* (Resende et al., 2012a; Resende et al., 2012b), this new method showed encouraging prospects, thus confirming the potential of GS in studies of conifers, pines, and eucalypts (Zapata-Valenzuela et al., 2013; Lima, 2014; El-Dien et al., 2015; Ratcliffe et al., 2015; Bartholome et al., 2016; Isik et al., 2016), which further supports the potential for GS to accelerate the breeding of forest trees.

In rubber tree breeding programs, pedigree-based analysis has been widely used to evaluate field experiments, estimate genetic parameters, and predict breeding values (Furlani et al., 2005). However, due to the decreasing costs of genotyping thousands or millions of markers and the increasing costs of phenotyping (Krchov and Bernardo, 2015), GS is emerging as an alternative genome-wide marker-based method to predict future genetic responses.

Genomic prediction models were originally developed for use in a single environment. However, to implement GS strategies in plant breeding, genotype × environment (G×E) interactions must be predicted. Habier et al. (2007) used genetic marker information to identify associations between individuals *via* the genomic relationship matrix K. Two very frequently used matrix-based methods include the genomic best linear unbiased predictor (GBLUP) (GB) (VanRaden, 2007, VanRaden, 2008) and the nonlinear Gaussian kernel (GK) methods (Gonzalez-Camacho et al., 2012). Burgueño et al. (2012) extended this general methodology to incorporate G×E effects. A separate GB extension introduces interaction effects between markers and environmental factors, and studies have shown that modeling G×E can result in substantial gains in PA (Heslot et al., 2014; Jarquin et al., 2014; Crossa et al., 2016; Cuevas et al., 2016).

A GBLUP model was proposed by Lopez-Cruz et al. (2015) to explicitly model the partitioning of genomic values and marker effects into components that are stable among environments and others that are environment specific. Therefore, according Cuevas et al. (2016), the marker × environment interaction model is suitable for application in groups of positively correlated environments. However, in practice, this approach can be very restrictive in cases where several environments have correlations close to zero, as it can lead to a large G×E variance component compared with the genetic variance component (Burgueño et al., 2011). VanRaden (2008) first suggested models in which the GBLUP was a linear model that included parameters associated with additive quantitative genetics theory.

A nonparametric and semiparametric method was proposed by Gianola et al. (2006) and accounted for small, complex epistatic interactions without explicitly modeling them. According to Heslot et al. (2012), the semiparametric reproducing kernel Hilbert space (RKHS method) uses a kernel function to convert the marker matrix into a set of distances between pairs of individuals. RKHS regression is thought to increase PA by capturing nonadditive variation, and several studies have confirmed this advantage (de los Campos et al., 2010; Perez-Rodriguez et al., 2013; Morota and Gianola, 2014).

Cuevas et al. (2016) applied GS with the marker × environment interaction model of Lopez-Cruz et al. (2015) and modeled the GB (linear kernel) and GK (nonlinear kernel) in a manner similar to that of de los Campos et al. (2010) in the RKHS with kernel averaging, and by estimating the bandwidth *via* an empirical Bayesian method (Pérez-Elizalde et al., 2015), and using wheat and maize data sets, they performed single-environment analyses and expanded them to account for G×E interactions. Compared with the other approaches, the GK combined with the G×E model provided greater flexibility and accounted for smaller, more complex marker main effects and marker-specific interaction effects (Cuevas et al., 2016). However, as in the study by Lopez-Cruz et al. (2015), this model assumes sets of environments that are positively correlated. To solve this problem, Cuevas et al. (2016) proposed two multienvironment genomic models to overcome some of the restrictions of previous genomic models.

Accurate predictions are obtained when the appropriate method is used even for untested genotypes, allowing considerable progress in breeding programs by reducing the number of field-tested genotypes and, consequently, the costs of phenotyping (Krchov and Bernardo, 2015). The benefits of GS are more evident when traits are difficult, time consuming, and expensive to measure and when several environments need to be evaluated.

The objective of this paper was to evaluate the predictive capability of GS implementation in rubber trees when linear and nonlinear kernel methods are used and to examine the performance of the predictions, including G×E interactions, of each of the four models described by Bandeira et al. (2017). Thus, for all data sets, we fitted models with a linear kernel *via* GB or GK with a bandwidth parameter estimated according to the methods of Pérez-Elizalde et al. (2015). We also compared the PA of the two kernel regression methods for the four models, which included the following: a single-environment, main genotypic effect model (SM); a multienvironment, main genotypic effect model (MM) (Jarquin et al., 2014); a multienvironment, single-variance G×E deviation model (MDs) (Jarquin et al., 2014); and a multienvironment, environment-specific variance G×E deviation model (MDe) (Lopez-Cruz et al., 2015).

To the best of our knowledge, this is the first attempt to apply GS with a multienvironment technique to a rubber tree breeding program. The development of a robust methodology enables the implementation of GS in routine evaluations to accelerate genetic progress.

## Materials and Methods

### Populations and Phenotypes

The data set included 435 samples, which comprised 252 F1 hybrids derived from a PR255 × PB217 cross (Souza et al., 2013; Rosa et al., 2018), 146 F1 hybrids derived from a GT1 × RRIM701 cross (Conson et al., 2018), 37 genotypes from a GT1 × PB235 cross, and 4 testers (GT1, PB235, RRIM701, and RRIM600), which are described further below.

#### Populations

The PR255 × PB217 population is a full-sib segregating population with a total of 252 individuals (progeny). Seedlings acquired *via* controlled pollination were clonally propagated by budding onto rootstocks. PR255 is a rapidly growing clone with vigorous and high yield, good growth, and stable latex production. In contrast, clone PB217 is the opposite, presenting slow growth and delayed latex production in its early years of development, although its latex production increases rapidly during the early years; however, this clone has potential for superior yield performance in the long term (Souza et al., 2013; Rosa et al., 2018). The field trial was performed in Itiquira, Mato Grosso state, Brazil (17°24′03″S and 54°44′53″W), from March 2006 until March 2007. The climate of this region is characterized by very dry and relatively cold winters and hot and humid summers, which represent conditions typical of southeastern Brazil, the most productive region for rubber. The experimental design was a randomized block design with four replications, with four grafted trees of the same individual in each plot (Rosa et al., 2018).

The GT1 × RRIM701 population comprised 146 individuals, and the GT1 × PB235 population comprised a total of 37 individuals. These two groups of progeny were derived from open pollination, and their effective pollination was checked *via* microsatellite markers. The hybrids were selected on the basis of polymorphisms between the parents. GT1 is a male-sterile clone that is classified as a primary clone (Shearman et al., 2014) and is tolerant to wind and cold. RRIM701 grows vigorously and presents an increased circumference after initial tapping (Romain and Thierry, 2011). PB235 is reported to be a high-yielding clone with an active metabolism and is known to be particularly susceptible to tapping panel dryness (Sivakumaran et al., 1988). These two groups of progeny were planted at the Center of Rubber Tree and Agroforestry Systems/Agronomic Institute (IAC) in the northwestern region of São Paulo state (20°25′00″S and 49°59′00″W at an altitude of 450 m), Brazil, in 2012 (Alvares et al., 2013). A modified block design was used (Federer and Raghavarao, 1975), and the trial was repeated four times. Each trial consisted of four blocks with two trees (clones) per plot spaced 4 m by 4 m. The experiment comprised a total of 656 (41 plots × 4 blocks × 4 replicates) plots and 1,312 trees (Conson et al., 2018).

#### Phenotypic Analysis

Stem circumference (SC, in cm) at 50 cm above ground level was measured to evaluate the growth of individual trees, where the average per plot was calculated. Growth traits were frequently measured only during the first 6 years, as height and SC are the main selection traits for rubber tree breeding (Rao and Kole, 2016). Measurements were taken at four different ages and are listed in **Supplementary Table 1**. Two sets of measurements were taken each year: one set applied to trees under low-water conditions (LW), and the other applied to trees under well-watered conditions (WW). These conditions were established according to the water distribution of each region in which the experiments were installed (**Supplementary Figure 1**, **Supplementary Table 2**).

Analyses of the SC traits were carried out *via* the breedR package (Munõz and Sanchez, 2017) in conjunction with the *remlf90* function and *method* = “*ai*,” and the best linear unbiased predictors (BLUPs) of each genotype used with the following mixed linear model were taken:

y=Xb+Zg+e

where y is the adjusted mean phenotypic value (best linear unbiased estimated [BLUES]), × and Z are known incidence matrices, b is the vector of fixed effects (environmental effects), and g is the vector of random effects (genetic effects). In the general model (*H* ^2^), when the entire data set from both environments (LW and WW) is used, the fixed effects included locale (place where the experiment was performed), block, water, and year. The G × E interaction and genotype were included as random effects in the model. When we considered each environment (LW or WW) separately $\left(H_{env}^{2}\right)$, the fixed effects were the G × E interaction, locale, year, repetition, and block. Genotype was included as a random effect.

The broad-sense heritability (*H* ^2^) (clonal mean heritability) was estimated for SC for each water management system (LW and WW) and for every data set:

$$H^{2}=\sigma_{g}^{2}/\left[\sigma_{g}^{2}+\frac{\sigma_{gxe}^{2}}{s}+\frac{e}{sa}\right]$$

where $\sigma_{g}^{2}$ is the genetic variance, $\sigma_{gxe}^{2}$ is the variance caused by the interaction between genotype and the environment, *e* is the residual variance, *s* is the number of environments, and *a* is the number of blocks.

For each environment, we estimated heritability $\left(H_{env}^{2}\right)$ separately as follows:

$$H_{env}^{2}=\sigma_{g}^{2}/\left(\sigma_{g}^{2}+\frac{\sigma_{e}^{2}}{r}\right)$$

where $\sigma_{g}^{2}\;$ is the genetic variance, $\sigma_{e}^{2}$ is the residual variance, and *r* is the number of trees per replicate.

### Genotypic Data and Single-Nucleotide Polymorphism Calling

Genomic DNA was extracted according to the methods of Souza et al. (2013) and Conson et al. (2018). Genotyping-by-sequencing (GBS) library preparation and sequencing were performed as described by Elshire et al. (2011). Genome complexity was reduced by digesting individual genomic DNA samples with *EcoT22I*, a methylation-sensitive restriction enzyme, and 96 samples were included in each sequencing lane. The resulting fragments from each sample were directly ligated to a pair of enzyme-specific adapters and combined into pools. PCR amplification was carried out to generate the GBS libraries. Library sequencing of GT1 × RRIM701 and GT1 × PB235 was performed on an Illumina GAIIx platform (Illumina Inc., San Diego, CA, United States), and sequencing of PR255 × PB217 was performed on the Illumina HiSeq platform.

The raw data were processed, and single-nucleotide polymorphism (SNP) calling was performed *via* TASSEL 5.0 (Glaubitz et al., 2014). Initially, the FASTQ files were demultiplexed according to their assigned barcodes. The reads from each sample were trimmed, and the tags were identified by the following parameters: Kmer length of 64 bp, minimum quality score within the barcode and read length of 20, minimum Kmer length of 20 and minimum count of reads for a tag of 6. The retained tags with a minimum count of six reads were aligned to the *H. brasiliensis* reference genome sequence (Tang et al., 2016) *via* Bowtie 2 version 2.1 (Langmead and Salzberg, 2012), with the *very sensitive* option enabled. SNP calling was performed *via* the TASSEL 5 GBSv2 pipeline (Glaubitz et al., 2014) and filtered with snpReady software (Granato and Fritsche-Neto, 2018). The following criteria were used: 20% missing data, minor allele frequency (MAF) greater than or equal to 5% (MAF of 0.05), and removal of individuals with more than 50% (*sweep.sample* = 0.5) missing data for the called SNPs. Only biallelic SNPs were maintained, which was performed *via* VCFtools (Danecek et al., 2011). After the data were filtered, the missing data were imputed by the *knni* method with snpReady software (Granato and Fritsche-Neto, 2018).

The genotypic data are available under NCBI accession PRJNA540286 (ID: 5440286) (GT1 × PB235 and GT1 × RRIM701) and accession PRJNA541308 (ID: 541308) (PR255 × PB217).

### Genomic Selection Analysis

Phenotypic analysis was carried out jointly for all years of evaluation *via* the mixed model approach.

Prediction based on genomic relationships and predictive ability assessment was performed *via* a relationship matrix-based approach for genomic prediction (Habier et al., 2007); the matrix K was the central object denoting the genomic relationship matrix. Two kernel methods were used: the linear kernel method (GB) used by Jarquin et al. (2014) and Lopez-Cruz et al. (2015) and the nonlinear kernel method (GK) proposed by Cuevas et al. (2016). The matrix for the GB (VanRaden, 2008) and GK (Gonzalez-Camacho et al., 2012) methods was obtained *via* the function *G.matrix* in snpReady software (Granato and Fritsche-Neto, 2018). Statistical models for genomic predictions taking G×E interactions into account (Jarquin et al., 2014; Lopez-Cruz et al., 2015) combine genetic information from molecular markers or from pedigrees (Pérez-Rodríguez et al., 2015) with environmental covariates, while the López-Cruz model breaks down the marker effect across all environments and the interaction for each specific environment.

The PA was obtained from the correlation between the predicted BLUPs and the observed BLUPs. Four statistical prediction models were fitted to all the data sets to study their PA *via* random cross-validation (CV) schemes. The main objective was to compare the prediction ability of kinship matrices (GB and GK) and the proposed single-environment and multienvironment (G×E) genomic models.

The PA values were also compared for the single-environment and multienvironment models SM, MM (Jarquin et al., 2014), MDs (Jarquin et al., 2014), and MDe (Lopez-Cruz et al., 2015) and fitted with the GB and GK methods; this was applied to all the data sets for all the studied traits. These analyses were performed to derive estimates of variance components resulting from the main genetic effect, and genetic environment-specific effects and residual effects of the four models described above for SC in the data sets from the two different conditions (LW and WW) were computed. For all GS models, BLUPs were estimated *via* the mixed model with breedR software (Munõz and Sanchez, 2017), and all models were fitted with G×E interactions *via* BGGE software (Granato et al., 2018), in which 20,000 iterations were performed (*ite* = 20,000), 5,000 samples were discarded (*burn-in* = 5,000), and every fifth iteration was used to estimate the posterior mean (*thin* = 5).

#### SM

Using the main effect of the genotype, the single-environment model fits data from each environment (LW and WW) separately. Equation (1) shows the matrix representation of this model.

(1) $$\;y=µ1+Z_{g}g+e$$

where *y* = (*y* *_1_* *…., y* *_n_*)’ is the response vector (BLUP), *y* *_i_* is the observation of the *i*th line (*i = 1,…., n*) in each environment, µ is the general mean, *Z* *_g_* is the incidence matrix that combines the random genetic effects and phenotypes, and *g* and *e* are the random genetic effect and the residual random effect, respectively, for each environment (LW and WW). In SM (1), *g* is considered to present a multivariate normal distribution with a mean of zero and a covariance matrix $\sigma_{gj}^{2}K$; that is, $\;g∼(0,\sigma_{gj}^{2}K)$, where $\sigma_{gj}^{2}$ is the genetic variance of *g* in the *j*th environment, and where *K* is a positive semidefinite symmetric matrix that shows the variance–covariance of the genetic values calculated from the molecular markers. Furthermore, the residual error *e* in each environment is considered to be separate from the homogeneous variance $\left(\sigma_{e}^{2}\right)$ and is distributed as $e\sim N\;(0,\sigma_{ej}^{2}I)$, where *I* is the identity matrix and $\sigma_{ej}^{2}\;$ is the residual variance in the *j* *^th^* environment. Thus, *g* is an estimation of the true unknown genetic values, and *e* includes the residual genetic effects that are not elucidated by *g* more other nongenetic effects that approximate the errors, as described by Bandeira et al. (2017). For SM (1), matrix K can be constructed using the linear kernel $K=\left(\frac{XX^{\prime }}{p}\right)$ (de los Campos et al., 2012) proposed by VanRaden (2007, 2008) for estimating the GBLUP, where x is the standardized matrix of molecular markers for the individuals, of order *n x p* and *p* is the number of markers. The entries of the GK are computed as $K\left(x_{i}x_{i`}\right)=\text{exp}(-hd_{ii`}^{2})$, where *d* *_ii_*_′_ is the Euclidean distance between the *i*th and *i* *^′th^* individuals $\left(i=1,\ldots ,\;n_{j}\right)\;$ given by the markers and *h* > 0 is the bandwidth parameter that controls the rate of decay of K values (Pérez-Rodríguez et al., 2013; Cuevas et al., 2016). In this study, GK takes the form $K\left(x_{i}x_{i`}\right)=\exp(-hd_{ii`}^{2}/median\left(d_{ii`}^{2}\right))$, where *h* = 1 and where the median of the distances is used as a scaling factor (Crossa et al., 2010). de los Campos et al. (2010) described the theory of the GK in the context of the RKHS KA (KA is well known as the GK, which is based on the Euclidian distance, aiming to capture additive and nonadditive effects).

#### MM

The MM takes into account the main fixed effects of environments, even in the presence of random genetic effects across environments. Equation (2) indicates the matrix representation of this model.

(2) $$y=µ1+X_{E}\beta_{E}+Z_{g}g+e$$

where *y* = (*y* *_1_* *, …, y* *_j_*,*…, y* *_s_*)’ is the response vector and *y* *_j_* is the vector of line observations (*i = 1,…, n* *_j_*) in the *j*th environment (*j = 1,…, s*). The fixed environmental effects in the data are models in the *X* *_E_* incidence matrix, where the intercept for each environment (β*_E_*) is the parameter to be estimated. The incidence matrix *Z* *_g_* is the other fixed effect that can be incorporated into the model, the matrix *Z* *_g_* combines genotype with phenotype for each environment, and *g* is the variance in the main genetic effects across environments. The random vector of genetic effects *g* across environments is considered to follow a multivariate normal distribution with a mean of zero and a covariance matrix of $\sigma_{g0}^{2}K$; that is, *g* ∼ $N(0,\;\sigma_{g0}^{2}K)$, where $\sigma_{g0}^{2}$ is the variance of the main genetic effects across environments and $e\sim \;N(0,\sigma_{e}^{2})$, as described by Bandeira et al. (2017). We used *g* with GB or GK.

#### MDs

The MDs extends the MM to implement the random interaction effect of the environments to incorporate more genetic information of the lines (*ge*).

(3) $$y=µ1+X_{E}\beta_{E}+Z_{g}g+ge+e\;\;$$

The vector of random effects of the interaction *ge* is considered to follow a multivariate normal distribution, ge~ N(0, [ZgKZg']∘[XEXE′]σge2. Here, (∘) is the Hadamard product operator and indicates, according to Jarquin et al. (2014), the product (element to element) between two matrices in the same order, and $\sigma_{ge}^{2}$ is the variance component of the interaction. The *K* matrix is defined the same as that above, and the vector of the main genetic effects is *g*, which presents a multivariate normal distribution with a mean of zero and a covariance matrix $\sigma_{g0}^{2}K$; that is, $g\sim N(0,\sigma_{g0}^{2}K)$, with variance of the main genetic effects $\left(\sigma_{g0}^{2}\right)$ and $e\sim (0,\sigma_{e}^{2}I)$, as described by Bandeira et al. (2017):

$$[Z_{g}KZ_{g}^{\prime }]\circ [X_{E}X_{E}^{\prime }]=\left[\begin{array}{cc} \begin{array}{cc} K_{1} & \cdots \\ \vdots & \ddots \end{array} & \begin{array}{cc} 0 & \begin{array}{cc} \cdots & 0 \end{array}\\ \vdots & \begin{array}{cc} \ddots & \vdots \end{array} \end{array}\\ \begin{array}{cc} 0 & \cdots \\ \begin{array}{c} \vdots \\ 0 \end{array} & \begin{array}{c} \ddots \\ \cdots \end{array} \end{array} & \begin{array}{cc} K_{j} & \begin{array}{cc} \cdots & 0 \end{array}\\ \begin{array}{c} \vdots \\ 0 \end{array} & \begin{array}{cc} \begin{array}{c} \ddots \\ \cdots \end{array} & \begin{array}{c} \vdots \\ K_{m} \end{array} \end{array} \end{array} \end{array}\right]$$

where *K* *_j_* represents the kernel constructed from the molecular markers of the lines in the *j*th environment. As in the MM, the matrix *K* is used in the variance–covariance for *g* of the MDs and is also a component of the variance–covariance of *g* *_e_*. The kernel matrix *K* can be constructed with GB and GK.

#### MDe

In the MDe (Lopez-Cruz et al., 2015), the genetic effects of markers are partitioned into main marker effects across all environments and specific marker effects within each environment. Equation (4) indicates the matrix representation of this model:

(4) $$y=µ1+X_{E}\beta_{E}+Z_{g}g_{0}+g_{E}+e$$

where *g* *_0_* denotes the main effect of the markers with a variance–covariance $g_{0}\sim N(0,\sigma_{g_{0}}^{2}K)$ across all environments, $\sigma_{g_{0}}^{2}$ is common to all *s* environments, and the borrowing of information among environments is generated through the kernel matrix K. Otherwise, the specific effect of the markers $\left(g_{E}\right)$ in environments or even the effects of the interaction with a variance–covariance structure differ from those of model (4); in other words, $g_{E}\sim N(0,K_{E})$, where *K* *_E_* is as follows:

KE=[σgE12K⋯0⋯0⋮⋱⋮⋱⋮0⋯σgEj2Kj⋯0⋮⋱⋮⋱⋮0⋯0⋯σgEj2Km]KE=[σgE12K⋯0⋯0⋮⋱⋮⋱⋮0⋯0⋯0⋮⋱⋮⋯⋮0⋯0⋯0]+⋯+[0⋯0⋯0⋮⋱⋮⋱⋮0⋯σgEj2Kj⋯0⋮⋱⋮⋱⋮0⋯0⋯0]+⋯+[0⋯0⋯0⋮⋱⋮⋱⋮0⋯0⋯0⋮⋱⋮⋱⋮0⋯0⋯σgEs2Ks]

The matrix *K* *_E_* can be expressed as the sum of *s* matrices, and the effects given by *g* *_Ej_* are specific for the *j*th environment, which has a variance–covariance matrix of $\sigma_{gEj}^{2}K_{j}$. These two terms (*g* and *g* *_E_*) of the MDe are given by the components of the estimated variance for the data. The kernel matrix *K* is used in the components of *g*, while kernel matrix *K* *_E_* is used in the component of *g* *_E_*; both *K* and *K* *_E_* can be used with GB or GK, as described by Bandeira et al. (2017).

### Assessing Prediction Accuracy by Random Cross-Validation

The PA of SM-method combinations was evaluated with the TRN set (which comprised 80% of the hybrids). The TST set comprised 20% of the individuals, and none of the lines to be predicted in the TST set were also in the TRN set, in which 5 random partitions were arranged 5-fold, with 100 random partitions each. This procedure was performed separately for each environment, namely, LW and WW, and the SMs were fitted separately for each environment.

The PA values of the multienvironment (LW and WW) model-method combinations were generated using two different cross-validation (CV) designs according to the methods of Burgueño et al. (2012). The random CV 1 design (CV1) assumes that new genotypes have not been tested or evaluated in either environment, where 20% of genotypes were not phenotyped in any environment and had to be predicted. The random CV 2 design (CV2) is a simulation of genotypes that has been evaluated in some environments but not in others. The CV2 design can be used only for multienvironment modeling methods (MM, MDs, and MDe) and not for single-environment (SM) modeling methods where the random CV is CV1.

The parameters of the models, which include the main genetic effects, variance components resulting from residual effects, G×E interaction effects, and environment-specific effects, were reestimated from the TRN data in each TRN-TST partition (50 random), and the models were fitted to the TRN data set. PA was assessed by computing Pearson’s product-moment correlations between predictions and phenotypes in the TST data set within environments.

### Expected Genetic Gain

Expected genetic gain (EGG) was estimated in two ways: the classic method used in rubber tree breeding *via* the breeder’s equation and phenotypic data and with information from the SNPs obtained *via* GS. The EGG was calculated according to the methods of Matias et al. (2019) and Grattapaglia (2017).

#### Expected Genetic Gain Obtained by a Classic Breeding Cycle With Only Phenotypic Information Used

The EGGs obtained by a classic breeding cycle (EGGcs) were estimated under the assumption that the time for first selection is 10 years. In rubber tree breeding, 3 years are needed from pollination to planting in the field, and as rubber trees usually require 6 years or more to reach tapping girth, there is a wait time of 7–9 years until tapping is started and a long period of 10 to 15 years before production and adaptation can be evaluated in the field (Gonçalves et al., 2005) according to the following equation:

$$EGGc=\frac{rc.i.\delta \text{g}}{T}$$

where rc is the accuracy of selection, in which the breeding improvement is equivalent to the square root of the *H* ^2^, *i* is the intensity selection, δg is the additive genetic standard deviation, and *T* is the selection cycle time.

#### Expected Selection Gain *via* Molecular Marker Information

The simulation of breeding cycles in which GS was used was based on the EGG *via* molecular marker information (EGGgs) equation, assuming a time of 3 years for each selection cycle and representing the time required for crossing, seed selection, and selection of the best individuals *via* molecular markers. The equation is as follows:

$$EGGgs=\frac{rgs.i.\delta \text{g}}{T}$$

where *rgs* is the selection accuracy with GS $\left(\frac{PA}{\sqrt{H^{2}}}\right)$, *i* is the intensity selection, δg is the additive genetic standard deviation, and *T* is the selection cycle time.

## Results

### Single-Nucleotide Polymorphisms Calling

We started with 435 genotypes, but three genotypes were replicates and thus were merged. We removed 27 individuals that had more than 50% missing SNPs, leaving 411 genotypes. After the data were analyzed, a total of 259.224 million reads of sequence data were obtained, of which 69.8% were high-quality barcoded reads. The overall alignment rate of these reads to the rubber tree reference genome (Tang et al., 2016) was 83.7%, and 23.1% were aligned exactly one time.

A total of 107,294 SNPs were identified. After markers 1) with more than 20% missing data, 2) with an MAF ≤ 0.05, 3) with more than two alleles were excluded, tags with a minimum depth of six reads were aligned to the *H. brasiliensis* reference genome sequence (Tang et al., 2016). This method was based on that of previous studies of other species, in which the authors argued that, compared with high-depth sequencing, low-depth (approximately 2–4X) sequencing enables more individuals to be genotyped for the same cost, which, according to Li et al. (2011), is a good strategy for genome-wide association studies (GWAS). Gorjanc et al. (2015) obtained similar results for GS studies and reported that optimal PA was obtained *via* low-depth sequencing (approximately 1–2X) of many genotypes.

After the data were filtered, 6.7% were missing. The mean depth ranged from 444 to 521 for GT1 × RRIM701 and GT1 × PB235, respectively, and was 202 for PR255 × PB217 (**Supplementary Figure 2**). Although large variation was observed between populations, only SNPs with at least six reads were selected, and the entire data set was reduced to 30.546 SNPs.

### Estimates of Genetic Parameters by Single-Nucleotide Polymorphism Genotyping

Using the genotyped SNPs, we assessed the population structure *via* principal component analysis (PCA), and the results indicated that the 411 genotypes fell into two major clusters (**Supplementary Figure 3**), which mainly contained hybrids derived from the PR255 × PB217 cross and hybrids derived from the GT1 × RRIM701 and GT1 × PB235 crosses. The first two PCs explained 19.5 and 2.2% of the total variance, respectively, clearly splitting the groups along the x- and y-axes.

### Descriptive Statistics

Box plots of SC in each environment are depicted in **Supplementary Figure 4**. The distribution of this trait in the environments was symmetrical (data not shown). The LW environment exhibited relatively high increases in SC, while the WW environment exhibited relatively low increases (**Supplementary Figure 4**). The trees grew better under increased water availability (WW) (data not shown); however, because the phenotypic measurements were taken twice per year for each tree, the phenotypes of the trees under WW were always measured at the beginning of the year, whereas the phenotypes of the trees under the LW were taken half a year later. This method inevitably generates a small difference between the two phenotypes because the trees under LW are older than those under WW when the same measurements are taken. Because rubber populations require extensive field trial planting, it is not feasible for a breeding program to maintain two planting areas and to examine two hydric conditions with trees of the same age.

To assess how much of the phenotypic variation is genetically controlled and thus appropriate for GS, we first estimated the *H* ^2^ of SC. The $H_{env}^{2}\;$ ranged from 0.51 to 0.50 for LW and WW, respectively (**Table 1**). The populations PR255 × PB217, GT1 × RRIM701, and GT1 × PB235 were evaluated in different environments that presented different site indexes (soil and climate conditions). The phenotypic variance differed between the sites, but we had common check genotypes in both environments; these checks served the connection between populations and other factors. Furthermore, on the basis of the results of heritability and residual distribution, it is evident that this approach allowed a reliable estimate of the error, factor effects, and their interactions.

**Table 1:** Phenotypic variation: heritability (*H* ^2^), variance genotype × environment (G×E) interaction $\left(\sigma_{gxe}^{2}\right)$, residual variance $\left(\sigma_{e}^{2}\right)$, genetic variance main effect $\left(\sigma_{g}^{2}\right)$, and coefficients of experimental variation (CVe%s) in environments with low-water conditions (LW) and with well-watered conditions (WW) considered together and alone, with p < .01 indicated by **.

	General	LW	WW
$\sigma_{g}^{2}$	3.61**	4.33	3.69
$\sigma_{gxe}^{2}$	0.81	–	–
$\sigma_{e}^{2}$	16.15	16.75	14.75
*H* ^2^	0.60	0.51	0.50
CVe%	20.00	20.40	19.10

On the basis of the phenotypic data, the estimates of genotypic variance $\left(\sigma_{g}^{2}\right)$ and G×E interaction variance $\left(\sigma_{gxe}^{2}\right)$ were relatively high (3.61) and relatively low (0.81), respectively, and both were significant. Under LW, $\sigma_{g}^{2}$ (4.33) was greater than that under WW (3.69). Similarly, the residual variance $\left(\sigma_{e}^{2}\right)$ estimate was greater under LW (16.75) than under WW (14.75). The coefficients of experimental variation (CVe%s) (**Table 1**) presented an overall value of 20%, ranging from 20.4% (LW) to 19.1% (WW), and were considered moderate.

### Estimates of the Variance Components

The estimates of the variance components for each of the GS models derived from the full data analysis are presented in **Table 2**.

**Table 2:** Estimates of different variance components for the following genomic selection (GS) models: the single-environment, main genotypic effect model (SM); the multienvironment, main genotypic effect model (MM); the multienvironment, single-variance genotype × environment (G×E) deviation model (MDs); and the multienvironment, environment-specific variance G×E deviation model, with the genomic best linear unbiased predictor (GBLUP, GB) and Gaussian kernel (GK) for stem circumference (SC).

	SM				MM		MDs		MDe			
	GK		GB		GK	GB	GK	GB	GK		GB	
	WW	LW	WW	LW	–	–	–	–	LW	WW	LW	WW
$\sigma_{g}^{2}$	0.47	0.50	0.44	0.46	0.96	0.99	0.93	0.97	0.80		0.90	
$\sigma_{e}^{2}$	0.53	0.50	0.56	0.54	0.04	0.01	0.02	0.01	0.02		0.01	
$\sigma_{gxe}^{2}$	–	–	–	–	–	–	0.05	0.02	–	–	–	–
$\sigma_{env}^{2}$	–	–	–	–	–	–	–	–	0.11	0.07	0.06	0.03

The genetic variance $\left(\sigma_{g}^{2}\right)$, residual variance $\left(\sigma_{e}^{2}\right)$, mean environmental genetic variance $\left(\sigma_{gxe}^{2}\right)$, and environment-specific genetic variance $\left(\sigma_{env}^{2}\right)$ are shown.

The variance components of genetic effects were greater when the GB method was used rather than when the GK method was used in all environments for the SM. Both the genetic variance and the environmental variance were greater when analyzed in the LW (**Table 2**). The residual variance for SM-GB was lower than that for SM-GK in all environments.

Compared with exclusion of the interaction term (G×E), inclusion of the term when the MM, MDe, and MDs were used led to a more significant reduction in the estimated residual variance, and for all the environments, the residuals from the GK were smaller than the residuals from the GB for the MM, MDs, and MDe. However, the multienvironment model (LW *versus* WW) assumed that there was no marker-environment interaction between families tested at the different sites and that there could be an effect between marker effect estimations from families tested at different sites and environments (LW and WW). This should be taken into consideration and should be carefully analyzed in the GK approach when additive *vs*. additive epistasis is targeted.

The residual variance components of MM-GK (corresponding to 4% of the total variance) and MM-GB (corresponding to 1% of the total variance) were similar; the genetic variance corresponded to 99% for MM-GB and 96% for MM-GK. The percentage of total variance corresponding to variance components of the genetic main effects of MDs-GK (93%) and MDe-GK (80%) was consistently smaller than that of the genetic main effects of MDs-GB (97%) and MDe-GB (90%) (**Table 2**). These results indicate that the G×E model (MM, MDe, or MDs) fits the data better than do single-environment models.

### Assessment of Prediction Accuracy

**Figure 1:**
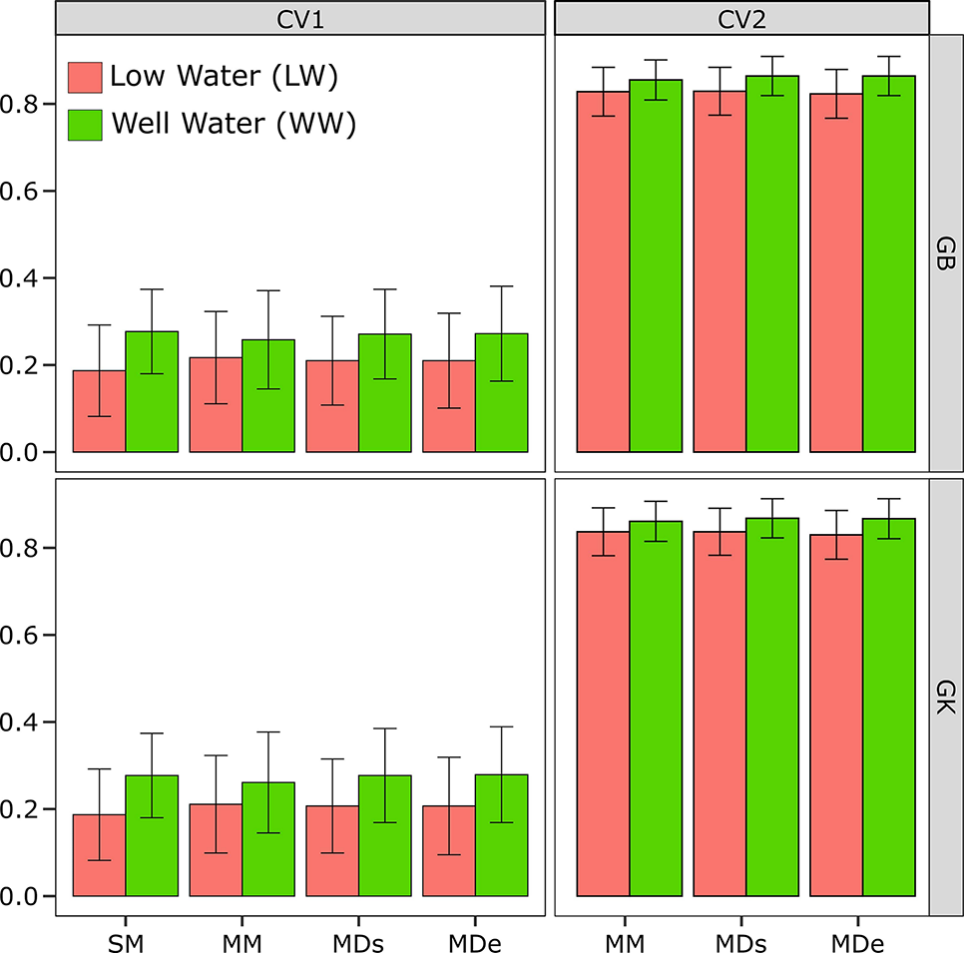
Correlations between phenotypes and prediction values for the single-environment, main genotypic effect model (SM) with the genomic best linear unbiased predictor (GBLUP) kernel method (SM-GB) and with the Gaussian kernel (GK) method (SM-GK); multienvironment, genotypic effect model with the GBLUP kernel (MM-GB) and with the GK (MM-GK); multienvironment, single-variance G×E model with the GBLUP kernel (MDs-GB) and with the GK (MDs-GK); and multienvironment, environment-specific variance G×E model with the GBLUP kernel (MDe-GB) and with the GK (MDe-GK) for stem circumference (SC). The environments included one with low-water conditions (LW) and one with well-watered conditions (WW).

The estimated correlations between the phenotypes and predictions obtained from the CV test are shown in **Figure 1** for the single-environment model (SM) and the multienvironment models (MM, MDs, and MDe).

#### Single-Environment Model (SM)

The CV2 design can be used only for multienvironment modeling methods (MM, MDs, and MDe) and not single-environment modeling methods (SM). Therefore, a single environment (SM) is analyzed, the random CV is CV1, but it is applied to only one individual environment (LW or WW) (**Figure 1**).

The results showed that the PA of the SM-GK combination was greater than that of the SM-GB combination under both LW and WW. The SC results were 0.19 under LW for SM-GB and 0.19 for SM-GK, and under WW, the results were 0.27 for SM-GB and 0.28 for SM-GK.

#### Multienvironment Models (MM, MDe, and MDs)

In terms of evaluating the PA of a model based on the correlation between the observed and the predicted values, when the PAs obtained by implementing different models (MM, MDs, and MDe) were compared, all the models were most accurate when CV2 was applied. The PA varied considerably between the CV1 and CV2 conditions (**Figure 1**). When only a random CV2 was considered, the PA results were very similar and varied little between environments.

The PA varied very little between the WW and LW (**Figure 1**). The results obtained with the model-method combinations were very similar. Generally, under LW, the best model was the GK, which did not differ between the methods (0.84), and MM-GB exhibited similar results (0.82). Relatively low PA values were obtained using the GB; the PA was 0.82 for the MDs and 0.83 for the MDe (**Figure 1**). Under WW, the model-method combinations presented the same values; the PA ranged from 0.86 for the MM to 0.87 for the MDe and MDs with both GK and GB (**Figure 1**).

### Expected Genetic Gain

The investigated alternative rubber tree breeding strategies differed considerably in the number of years required to finish one breeding cycle. For the classic improvement strategy, we considered a minimum duration of 10 years for the beginning of the selection of the best genotypes because 3 years are required from pollination to planting in the field and because rubber trees generally require several additional years (often 6 or more) to reach tapping girth. In the case of GS, we considered 3 years for initial selection (from pollination to field planting).

**Figure 2:**
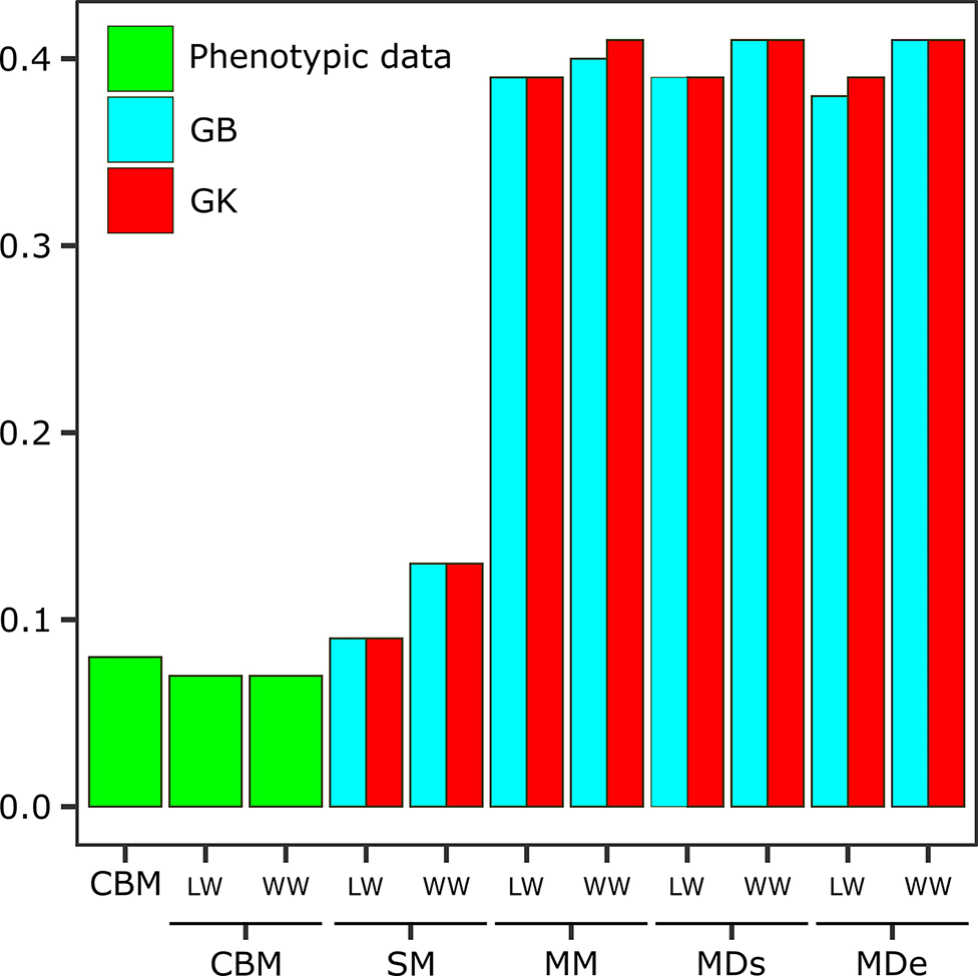
Expected genetic gain (EGG) obtained *via* the classic breeding method (CBM) with phenotypic data sets and analyzed in separate environments [one with low-water conditions (LW) and one with well-watered conditions (WW)] and EGG obtained *via* the following genomic selection (GS) models: the single-environment, main genotypic effect model (SM); multienvironment, genotypic effect model (MM); multienvironment, single-variance G×E model (MDs); and multienvironment, environment-specific variance G×E model (MDe), with GB and GK shown in the two evaluated environments (LW and WW).

The EGG calculations were performed for all the methods and models and were compared with classic improvements in both environments (**Figure 2** and **Supplementary Table 3**). When the CBM, which takes into account only phenotypic data, was used, the selection gain without considering the environment was 0.08, and if the data were separated by environment, the EGGc was the same (0.07) for LW and WW (**Figure 2**). When we incorporated genotypic information in a single environment (SM), the genetic gain increased to 0.13 for the WW when GB was used and 0.09 when GK was used, while for LW, there was no difference between GK and GB.

The genetic gains obtained when the information from the G×E interaction was incorporated were much greater gains than those obtained from a single environment. However, the results varied little between methods, with similar values resulting from most of the analyses. Considering the overall LW, the EGG was 0.39 for all models and methods except MDe-GB (0.38). For WW, the EGG was slightly greater than that under LW, and most models and methods had estimated gains of 0.41, with the exception of MM-GB (0.40) (**Figure 2**).

## Discussion

Incorporating and improving the genomic PA of rubber trees are a challenge for the successful application of GS in breeding programs. In this research, genomic PA was studied in rubber tree data sets *via* the GB and GK methods in conjunction with multienvironment models that evaluated trees under contrasting hydric conditions in different seasons of the year (LW and WW).

Many factors such as genetic architecture, heritability, population structure, and marker density can influence GS (Crossa et al., 2017). According to Meuwissen et al. (2001), GS is expected to increase the accuracy of selection, particularly for traits that have a low heritability and that cannot be measured directly from breeding candidates.

The accuracy of GS also depends on the genetic architecture of traits, such as heritability which are positively related to PA. Complex traits that present low heritability and small marker effects are suitable for GS. Our analyses revealed moderate heritability estimates for SC ranging from 0.50 to 0.51, with the lowest value for WW and the highest for LW (**Table 1**). Nevertheless, the heritabilities estimated in this study were within the range of those estimated in other studies for SC in *Hevea*, which were *H* *^2^* = 0.32 (Moreti et al., 1994) and *H* *^2^* = 0.47 (Gonçalves et al., 1999).

The CVe% for SC (**Table 1**) ranged from 20.4% (LW) to 19.1% (WW), which is considered moderate according to the classification proposed by Costa et al. (2010), who described the coefficient of variation as a useful tool to efficiently and accurately specify the experimental results: the lower the CVe% is, the more homogeneous the data, and the less environmental interference. The environmental variation, genotypes, and interaction between these two factors were highly significant, indicating that the environments used were contrasting, that there was genetic variability among the genotypes, and that the genotypes performed differently depending on the environment.

Using the CBM, Moreti et al. (1994) estimated genetic parameters and expected gains *via* the selection of juvenile characters in rubber tree progeny, and some parameters (rubber production, bark thickness, and SC) positively stood out. Gonçalves et al. (1996) observed the same phenomenon in the results reported by Moreti et al. (1994), showing a correlation and its applicability to the selection process. Strong phenotypic and genetic correlations were observed between yield and SC, indicating the possibility of obtaining young clones of good productive capacity and high vigor (Gonçalves et al., 1984). This correlation in conjunction with moderate heritability could be used to perform early selection of more productive clones without the need to wait for the trees to enter production, which requires an extended evaluation period.

Trees with rapid SC development may be more productive, and this feature may be a useful way to predict more productive hybrids *via* GS. Given this and latex production having greater heritability than circumference growth because the influence of the rootstock is relatively low in production, this feature will be very important in future studies of this population.

In GS, G×E interactions can be modeled by a marker × environment interaction and by a linear kernel or a nonlinear GK (Cuevas et al., 2016). Multienvironment genomic prediction was successfully implemented using a GBLUP model; however, depending on the genetic architecture of the trait and germplasm, nonlinear semiparametric approaches such as GK could produce more accurate results than could linear approaches (Cuevas et al., 2016).

Here, the GK methods presented a small increase in the prediction ability of all single-environment and multienvironment models with CV2, confirming the results of Lopez-Cruz et al. (2015) and Zhang et al. (2015) and demonstrating that predicting new genotypes is more complicated than predicting genotypes that have been evaluated in correlated trials. The GB method was superior when analyzed only *via* CV1 under LW.

The multienvironment models and the GK method resulted in the best PA. Similar decreases in PA were reported by Lopez-Cruz et al. (2015) when wheat lines were used and by Bandeira et al. (2017) when a maize data set was analyzed in attempts to predict lines in untested environments under a CV1 random partitioning scheme.

Considering only random CV2, the PA was slightly greater under WW, ranging from 0.87 (MDe-GK-WW) to 0.82 (MDe-GB-LW), which is consistent with previously published results for forest tree species. Bartholome et al. (2016) reported medium to high PAs for all traits studied (0.52 to 0.91) in maritime pine. Similar accuracies were reported for the height of loblolly pine trees, with values ranging from 0.64 to 0.74 (Resende et al., 2012b), and eucalyptus hybrids (0.66 to 0.79) (Resende et al., 2012a), regardless of differences in GS models, species, and population structure between studies.

If information concerning WW and LW was combined with multienvironment models, the results were superior to those of single-environment genomic models with GB and GK. This finding suggests that introducing interactions between markers and environmental conditions can increase the proportion of variance accounted for by the model and, more importantly, can increase the PA. Optimized crosses *via* selection of the best stable parents can then be performed to improve hybrid stability and the EGG (Toro and Varona, 2010).

G×E interactions are essential in many aspects of a breeding program, and the increase in PA with the inclusion of environmental information represents a favorable result with important implications for both breeding and agronomic recommendations. In rubber tree breeding, progeny testing is commonly used to evaluate the performance of new genotypes. Thus, in this case, new hybrids identified as high-performance hybrids with stable development throughout the year can be selected for use in new biparental crosses or new population selections. Interactions in field trials affect both early selection and mature selection; therefore, when the effectiveness of early selections is evaluated, it is important to determine whether the G×E interactions among environments significantly affect the genetic correlation of early maturity.

Application of the combinations of four models (SM, MM, MDs, and MDe) and two kernel methods (GB and GK) to rubber tree data sets revealed that the PAs of the models with the nonlinear GK were similar to those of the models with the linear GB kernel. According to Gianola et al. (2014), the GK has a better predictive ability and a more flexible structure than does the GB, and the GK can capture nonadditive effects between markers.

Akdemir and Jannink (2015) presented different choices for estimating kernel functions: linear kernel matrices incorporate only the additive effects of the markers, polynomial kernels incorporate different degrees of marker interactions, and the GK function uses complex epistatic marker interactions. GK would be more appropriate for GS of rubber trees because of the possibility of exploiting the local epistatic effects captured in the GK and their interactions with environments.

Many GS studies of plants have focused on breeding programs that generally evaluate crops in multiple environments, such as in different seasons/years or in geographic locations, to determine performance stability across environments (Crossa et al., 2016) and to identify markers whose effects are environment specific or whose effects are stable across environments (Crossa et al., 2016; Oakey et al., 2016). Previous studies in wheat (Lopez-Cruz et al., 2015) expanded the single-trait GB model to a multienvironment context and revealed substantial gains in PA with the multienvironment model compared with the single-environment model.

Advantages of GS applied to the improvement of forest species have been demonstrated. For example, Wong and Bernardo (2008) and Iwata et al. (2011) demonstrated the potential uses of GS and concluded that it could dramatically increase tree breeding efficiency. The advantage of marker-based relationship matrices is that gaps in pairwise relatedness in forest tree pedigrees are filled, which leads to increased accuracy of breeding candidate selection (Muller et al., 2017; Tan et al., 2017).

Using both genetic markers and environmental covariates, Cuevas et al. (2016) modeled G×E interactions, and Granato et al. (2018) introduced the Bayesian Genomic Genotype × Environment (BGGE) R package, which fits genomic linear mixed models to single environments and multiple environments with G×E models. These studies showed that modeling multienvironment interactions can lead to substantial gains in the PA of GS for rubber tree breeding programs.

GS is expected to increase the accuracy of selection, especially for traits that cannot be measured directly from breeding candidates and for traits with a low heritability (Meuwissen et al., 2001), and this effect was confirmed in the present study. The selection gain with GS for SC was on average 0.40, while the genetic gain with the CBM was 0.08. When the CBM for rubber trees was compared with the GS method while the multienvironment strategy was applied (MM, MDe and MDs), GS resulted in a five-fold greater genetic gain for SC.

### Implementing Genomic Selection in Rubber Tree Breeding Programs

In the last decade, many statistical models have been proposed for applying GS in plant and animal breeding programs and have received increasing interest from forest tree breeders. Resende et al. (2012a, 2012b) demonstrated encouraging prospects of this new method, and the potential for GS in conifers, pines, and eucalypts has since been confirmed (Zapata-Valenzuela et al., 2013; Lima, 2014; El-Dien et al., 2015; Ratcliffe et al., 2015; Bartholome et al., 2016; Isik et al., 2016), supporting further the potential for GS to accelerate the breeding of forest trees. In the case of rubber trees, a recent study explored GS in a breeding program (Cros et al., 2019).

In this study, we used three full-sib populations, taking advantage of breeding populations that had already been genotyped and phenotyped (Rosa et al., 2018; Conson et al., 2018). This type of population is favorable for GS because of the high LD between marker alleles and genetic alleles. Similar results were obtained in a recent study in which a biparental rubber tree population with 189 and 143 clones of the cross PB260 × RRIM600 was used; the population was genotyped with a limited number of markers (332 simple sequence repeat markers) (Cros et al., 2019), which resulted in a GS accuracy of 0.53. Other plant species have also been evaluated, with GS accuracies reaching moderate to high values (0.59 and 0.91) in a family of 180 *Citrus* clones (Gois et al., 2016).

For rubber trees, the time required to complete a breeding cycle and recommend a clone for commercial production can span multiple decades and is divided mainly into three selection stages. First, the aim is to obtain progeny by controlled or open pollination and to establish nurseries. At two and-a-half years, on the basis of early evaluations of yield, vigor, and tolerance to disease, breeding trees are selected and cloned for testing at a small scale. During this second stage of the selection cycle, after the first 2 years of tapping, promising clones are multiplied and subsequently evaluated in large-scale or regional trials. This last stage usually takes 12 to 15 years, until it is possible to recommend a clone for large-scale cropping. Therefore, it takes approximately 30 years to complete the breeding cycle, from controlled pollination to final cultivar recommendation (Gonçalves and Fontes, 2012).

In essence, implementing techniques that reduce the long breeding cycle of trees is urgently needed, and for this purpose, the use of a biparental population was a means of managing the difficulty of obtaining complex families, which can take many years to generate because of the low fecundity of trees and the long duration of the phenotypic evaluation needed. According to Cros et al. (2019), a GS approach in which a complex population involving several families is used could lead to variation in GS among selection candidates depending on their relationships with the TRN individuals, leading to GS accuracies lower than those from family-specific TRN populations.

In addition, large areas are required for the development of hybrids, which not only increases the costs associated with maintaining plants in the field but also limits the number of genotypes that can be evaluated. GS can minimize these difficulties because selection can be performed on juvenile plants, which reduces the interval between generations and increases the intensity of selection, thus reducing the gain per unit time (Resende et al., 2008; Wong and Bernardo, 2008; Heffner et al., 2009).

The use of GS could dramatically reduce the time required for completion of a genetic improvement cycle by eliminating phenotypic progeny testing aimed at selecting the best individuals (replaced by GS), significantly increasing the genetic gain relative to that obtained by CBMs. Another advantage of GS compared with phenotypic selection is that more candidate genotypes are generated; therefore, the population size for selection is improved. All of the candidates are genotyped, and those with the best-predicted test cross values are evaluated in the field; this process can be considered a form of indirect selection.

According to Heffner et al. (2009), even when only moderate accuracy is obtained with GS, it is possible to obtain a genetic gain greater than that obtained by phenotypic selection, as GS reduces the duration of the selection cycle. According to Wong and Bernardo (2008), the selection cycle was shortened from 19 to 6 years when GS was implemented in oil palm. Similar results were observed in the present study, in that the length of the selection cycle was also reduced.

With declining costs and rapid advances in genotyping methods, even with the costs of maintaining large progeny trials and the potential for increased gains per unit time, we very cautiously expect GS to have excellent potential for implementation in rubber tree breeding programs. However, additional studies examining populations with different structures (which were not assessed in this initial work) are necessary before recommending GS for operational implementation in tree breeding programs.

This is the first study to incorporate models for G×E interaction when phenotypic and/or genotypic information was used simultaneously for genetic prediction in the context of GS in a rubber tree breeding program. The results presented here suggest that GS can be useful for rubber tree breeding because this technique can be used to accurately predict the phenotypes and reduce the length of the selection cycle. Thus, GS is a promising tool for improving rubber tree cultivation, and we look forward to exploring the historical phenotypic data collected during 15 years as part of national breeding programs.

## Data Availability Statement

Publicly available datasets were analyzed in this study. This data can be found here: https://www.ncbi.nlm.nih.gov/bioproject/PRJNA540286 and https://www.ncbi.nlm.nih.gov/bioproject/PRJNA541308

## Author Contributions

LS, FF, PG, RF-N and AS designed the study and performed the experiments; VG and EJ performed the experiments in the field; LS, FF, and RF-N analyzed the data; LS, FF and AS wrote the manuscript. and VG and EJ performed the experiments in the field.

## Funding

The authors gratefully acknowledge the Fundação de Amparo a Pesquisa do Estado de São Paulo (FAPESP) for a Ph.D. fellowship to FF (18/18985-7); the Coordenação de Aperfeiçoamento do Pessoal de Nível Superior (CAPES) for financial support (Computational Biology Program and CAPES-Agropolis Program) and postdoctoral fellowships to LS (88887.334728/2019-00); and the Conselho Nacional de Desenvolvimento Científico e Tecnológico (CNPq) for financial support, a postdoctoral fellowship to LS (168028/2017-4), and research fellowships to AS and PG.

## Conflict of Interest

The authors declare that the research was conducted in the absence of any commercial or financial relationships that could be construed as a potential conflict of interest.
