# Supplementary material for: Genomic Selection in Rubber Tree Breeding: A Comparison of Models and Methods for Managing G×E Interactions
Source: Front Plant Sci. 2019 Oct 25;10:1353. doi: 10.3389/fpls.2019.01353 (PMC6824234; doi:10.3389/fpls.2019.01353)
Supplement: Supplementary file 1 [file DataSheet_1.pdf]

Supplementary Material

Supplementary Tables

Supplementary Table 1. Dates of the phenotyping of the two populations.

	Michelin		IAC	
Year	LW	WW	LW	WW
1	October/07	-	December/13	June/13
2	October/08	April/08	November/14	May/14
3	October/09	April/09	-	June/15
4	October/10	April/10	-	June/16

Supplementary Table 2. *BLUP* (Best Linear Unbiased Prediction) of stem circumference (SC, in cm) taken at four different ages. Two measurements were taken during each year: one was considered low-water (LW), and the other was considered well-watered (WW).

Genotype	SC	SC.se	SC-LW	SC.se-LW	SC-WW	SC.se-WW
1	0,022	0,703	-0,033	0,491	-0,083	0,577
10	3,824	0,733	4,107	0,57	3,699	0,667
100	-2,026	0,697	-2,828	0,476	-1,791	0,56
101	-0,356	0,697	-0,807	0,476	-0,192	0,56
102	1,324	0,697	1,194	0,476	1,361	0,56
103	1,981	0,693	2,116	0,463	1,986	0,544
104	-2,561	0,693	-3,104	0,463	-2,613	0,544
105	1,98	0,703	1,84	0,491	2,06	0,577
106	2,531	0,693	2,716	0,463	2,397	0,544
107	-0,221	0,716	-0,53	0,526	-0,229	0,617
108	1,686	0,864	1,531	0,845	1,725	0,965
109	-2,218	0,758	-2,688	0,629	-2,234	0,734
11	-1,853	0,701	-2,489	0,485	-1,709	0,571

110	1,632	0,693	1,667	0,463	1,614	0,544
111	1,237	0,697	1,251	0,476	1,095	0,56
112	-1,317	0,709	-1,936	0,507	-1,143	0,596
113	-0,026	0,722	-0,505	0,538	0,327	0,641
114	0,161	0,703	-0,03	0,491	0,068	0,577
115	2,055	0,703	1,858	0,491	2,243	0,577
116	5,174	0,693	5,978	0,463	4,826	0,544
117	0,749	0,693	0,994	0,463	0,421	0,544
118	2,006	0,697	1,897	0,476	2,085	0,56
119	3,908	0,716	4,243	0,526	3,855	0,617
120	-0,043	0,713	-0,17	0,526	-0,198	0,603
121	-4,808	0,825	-5,708	0,77	-4,306	0,887
122	-0,105	0,704	-0,374	0,497	-0,098	0,577
123	-0,999	0,722	-1,517	0,542	-0,817	0,633
124	-0,251	0,703	-0,492	0,491	-0,268	0,577
125	-1,818	0,703	-2,495	0,494	-1,65	0,577
126	-2,298	0,711	-3,02	0,511	-2,049	0,603
127	-3,371	0,703	-4,427	0,491	-3,083	0,577
129	-0,217	0,922	-0,477	0,948	-0,126	1,069
13	-0,696	0,693	-1,072	0,463	-0,738	0,544
130	-0,797	0,708	-1,168	0,507	-0,837	0,589
131	0,335	0,708	0,34	0,504	0,13	0,596
132	1,91	0,697	1,999	0,476	1,748	0,56
133	-0,082	0,697	-0,452	0,476	-0,01	0,56
134	0,688	0,693	0,521	0,463	0,657	0,544
135	-3,427	0,703	-4,462	0,491	-3,094	0,577
136	0,045	0,696	-0,057	0,473	-0,197	0,554
137	1,045	0,693	0,848	0,464	1,131	0,545
138	1,088	0,697	1,077	0,476	1,081	0,56
139	0,733	0,702	0,783	0,491	0,637	0,571
14	-0,808	0,693	-1,357	0,463	-0,628	0,544
140	-0,506	0,716	-0,898	0,526	-0,468	0,617
141	2,534	0,775	2,45	0,667	2,503	0,775
142	0,861	0,709	0,655	0,507	0,974	0,596
143	1,955	0,697	1,91	0,476	2,016	0,56
144	-0,838	0,697	-1,175	0,476	-0,786	0,56
145	-0,501	0,697	-0,864	0,476	-0,473	0,56
146	2,185	0,693	2,367	0,463	2,021	0,544
147	0,892	0,703	0,746	0,491	1,083	0,577
148	2,425	0,697	2,657	0,476	2,314	0,56
149	0,406	0,697	0,18	0,476	0,534	0,56
15	2,385	0,741	2,552	0,591	2,293	0,687
150	-0,611	0,703	-0,903	0,491	-0,496	0,577

151	0,841	0,693	0,816	0,463	0,656	0,544
152	-0,087	0,703	-0,36	0,491	-0,146	0,577
153	1,535	0,693	1,851	0,464	1,521	0,545
154	-1,507	0,698	-2,026	0,476	-1,521	0,56
155	0,052	0,697	0,022	0,476	-0,156	0,56
156	-5,391	0,693	-6,591	0,465	-5,142	0,544
157	-1,834	0,724	-2,656	0,546	-1,542	0,641
158	0,101	0,711	-0,236	0,511	0,389	0,603
159	-0,018	0,697	-0,556	0,476	0,3	0,56
16	0,21	0,697	0,025	0,476	0,356	0,56
160	4,292	0,697	4,829	0,476	4,082	0,56
161	3,758	0,703	4,096	0,491	3,586	0,577
162	1,4	0,724	1,512	0,546	1,189	0,64
163	1,833	0,699	1,765	0,479	2,049	0,565
164	0,518	0,697	0,294	0,476	0,697	0,56
166	0,609	0,693	0,245	0,463	0,764	0,544
167	2,363	0,693	2,69	0,463	2,113	0,544
168	0,83	0,703	0,54	0,491	0,987	0,577
169	-0,953	0,724	-1,513	0,546	-0,631	0,641
17	1,936	0,713	2,028	0,526	1,855	0,603
170	-3,724	0,694	-4,787	0,468	-3,496	0,549
171	1,526	0,721	1,052	0,538	1,951	0,633
172	-0,124	0,697	-0,325	0,476	-0,126	0,56
173	0,686	0,693	0,532	0,463	0,726	0,544
174	-1,23	0,698	-1,66	0,476	-1,059	0,565
175	-0,739	0,709	-1,246	0,507	-0,548	0,596
176	-1,464	0,697	-2,078	0,476	-1,194	0,56
177	3,282	0,693	3,527	0,463	3,231	0,544
178	1,802	0,708	1,847	0,508	1,752	0,59
179	-0,482	0,704	-0,741	0,494	-0,556	0,583
18	0,724	0,693	0,606	0,463	0,773	0,544
180	-0,781	0,698	-1,085	0,477	-0,809	0,56
181	-1,672	0,707	-2,374	0,504	-1,402	0,589
182	-0,087	0,693	-0,406	0,463	-0,104	0,544
183	-1,707	0,733	-2,266	0,57	-1,508	0,667
184	-5,248	0,693	-6,531	0,463	-5,039	0,544
185	-1,285	0,703	-1,895	0,491	-1,175	0,577
186	0,876	0,709	0,791	0,507	0,742	0,596
187	-2,107	0,709	-2,66	0,507	-1,998	0,596
188	-0,26	0,709	-0,703	0,508	0,189	0,596
189	0,588	0,693	0,23	0,463	0,758	0,544
19	0,724	0,697	0,801	0,476	0,591	0,56
190	-1,498	0,724	-2,123	0,546	-1,33	0,641
191	0,345	0,693	0,093	0,463	0,426	0,544

192	-0,64	0,699	-1,052	0,479	-0,504	0,565
193	-1,334	0,699	-1,655	0,479	-1,452	0,565
194	0,131	0,7	-0,085	0,488	0,064	0,565
195	1,604	0,693	1,709	0,463	1,455	0,544
196	1,763	0,696	1,769	0,473	1,705	0,554
197	0,969	0,697	0,91	0,476	0,857	0,56
198	2,651	0,716	2,818	0,526	2,565	0,617
199	-1,92	0,709	-2,618	0,508	-1,664	0,596
2	0,863	0,702	0,916	0,491	0,852	0,571
20	-0,284	0,697	-0,449	0,476	-0,501	0,56
200	0,364	0,694	0,251	0,465	0,292	0,549
201	-0,362	0,699	-0,719	0,479	-0,133	0,565
202	0,241	0,703	0,016	0,491	0,297	0,577
203	1,809	0,701	1,744	0,488	1,82	0,571
204	1,491	0,693	1,622	0,463	1,294	0,544
205	0,714	0,693	0,605	0,463	0,7	0,544
206	0,146	0,693	-0,006	0,463	-0,018	0,544
207	4,503	0,693	5,302	0,463	4,033	0,544
208	1,808	0,704	1,943	0,494	1,773	0,583
209	1,079	0,693	0,699	0,463	1,372	0,544
21	-1,064	0,716	-1,75	0,526	-0,764	0,617
210	-0,059	0,693	-0,463	0,463	0,036	0,544
211	-0,533	0,693	-0,879	0,463	-0,494	0,544
212	0,27	0,709	-0,022	0,509	0,618	0,598
213	0,233	0,693	0,305	0,463	0,094	0,544
214	-0,337	0,704	-0,953	0,491	0,015	0,583
215	1,105	0,693	1,045	0,463	1,061	0,544
216	-0,928	0,697	-1,384	0,476	-0,857	0,56
217	0,745	0,718	0,595	0,53	0,826	0,625
218	4,948	0,697	5,694	0,476	4,575	0,56
219	-1,999	0,825	-2,596	0,77	-1,625	0,887
22	-0,796	0,697	-1,089	0,476	-0,914	0,56
220	2,892	0,703	3,273	0,491	2,641	0,577
222	3,474	0,697	3,934	0,476	3,2	0,56
223	-0,122	0,703	-0,15	0,491	-0,191	0,577
224	4,428	0,703	4,968	0,491	4,21	0,577
225	2,354	0,693	2,372	0,463	2,373	0,544
226	0,946	0,693	0,842	0,463	0,865	0,544
227	-0,711	0,693	-1,056	0,463	-0,747	0,544
228	-3,344	0,693	-4,467	0,465	-2,999	0,544
229	-0,661	0,697	-1,33	0,476	-0,374	0,56
23	0,359	0,716	0,109	0,526	0,4	0,617
230	4,113	0,701	4,554	0,488	3,981	0,571

231	1,758	0,697	1,478	0,476	1,982	0,56
232	2,005	0,697	2,022	0,476	1,99	0,56
233	-2,077	0,706	-2,742	0,501	-1,923	0,583
234	-0,092	0,716	-0,33	0,526	-0,026	0,617
235	0,78	0,703	0,705	0,491	0,748	0,577
236	0,636	0,697	0,327	0,476	0,799	0,56
237	2,629	0,724	2,924	0,546	2,386	0,641
238	0,275	0,71	0,116	0,507	0,309	0,602
239	0,506	0,724	0,23	0,546	0,609	0,64
24	-2,333	0,697	-3,039	0,476	-2,26	0,56
240	-0,953	0,703	-1,229	0,491	-1,048	0,577
241	0,94	0,709	0,671	0,508	1,021	0,596
242	-2,175	0,701	-2,822	0,488	-2,133	0,571
243	0,201	0,703	-0,024	0,491	0,211	0,577
244	1,241	0,709	1,124	0,507	1,237	0,596
245	-0,078	0,705	-0,201	0,501	-0,279	0,583
246	1,101	0,697	1,048	0,476	1,039	0,56
247	2,15	0,694	1,99	0,463	2,492	0,549
248	0,095	0,697	-0,274	0,476	0,175	0,56
249	-2,854	0,725	-3,766	0,546	-2,445	0,649
250	-1,025	0,716	-1,419	0,527	-0,93	0,619
252	1,951	0,703	2,319	0,491	1,62	0,577
253	1,134	0,703	0,976	0,491	1,157	0,577
254	-0,208	0,709	-0,448	0,507	-0,156	0,596
256	-0,401	0,709	-0,866	0,507	-0,181	0,596
259	-1,588	0,702	-1,988	0,491	-1,606	0,571
26	-0,036	0,693	-0,4	0,463	0,069	0,544
27	1,267	0,697	1,251	0,476	1,172	0,56
270	2,298	0,703	2,597	0,491	2,035	0,577
28	0,308	0,72	0,075	0,542	0,462	0,625
29	-0,334	0,697	-0,748	0,476	-0,312	0,56
3	-0,65	0,693	-0,93	0,463	-0,796	0,544
30	0,86	0,703	0,749	0,491	0,766	0,577
31	1,215	0,693	1,094	0,463	1,248	0,544
32	2,085	0,703	2,375	0,491	1,716	0,577
33	2,82	0,697	3,164	0,476	2,567	0,56
34	0,999	0,697	0,935	0,476	0,927	0,56
35	3,597	0,703	3,969	0,491	3,462	0,577
36	-1,361	0,708	-1,959	0,507	-1,222	0,589
37	0,362	0,718	0,141	0,53	0,354	0,625
38	1,62	0,693	1,512	0,463	1,655	0,544
39	-1,63	0,698	-2,318	0,476	-1,35	0,565
4	2,54	0,698	2,697	0,477	2,645	0,56
40	-2,546	0,693	-3,42	0,463	-2,328	0,545

41	5,223	0,703	5,876	0,491	5,005	0,577
42	3,622	0,693	3,997	0,463	3,462	0,544
43	0,288	0,697	-0,191	0,476	0,526	0,56
44	0,574	0,697	0,343	0,476	0,609	0,56
45	-1,105	0,703	-1,423	0,491	-1,08	0,577
46	2,813	0,697	3,181	0,476	2,509	0,56
47	-1,968	0,697	-2,581	0,476	-1,977	0,56
48	-1,502	0,697	-2,098	0,476	-1,395	0,56
49	-0,854	0,703	-1,333	0,491	-0,687	0,577
5	2,913	0,709	3,47	0,508	2,675	0,596
50	-1,605	0,693	-2,221	0,463	-1,501	0,544
51	-0,494	0,697	-0,915	0,476	-0,329	0,56
52	-1,161	0,724	-1,647	0,546	-1,118	0,641
53	-1,305	0,709	-1,748	0,507	-1,2	0,596
54	0,815	0,696	0,566	0,473	0,904	0,554
55	-0,412	0,698	-0,508	0,479	-0,538	0,56
56	-0,773	0,693	-1,35	0,463	-0,572	0,544
57	3,793	0,722	4,33	0,547	3,371	0,633
58	-1,703	0,709	-2,101	0,507	-1,738	0,596
59	0,449	0,716	0,215	0,526	0,523	0,617
6	2,895	0,697	3,436	0,476	2,561	0,56
60	-0,658	0,714	-0,775	0,526	-0,767	0,61
61	2	0,716	2,073	0,526	1,823	0,617
62	-1,257	0,709	-1,797	0,507	-1,21	0,596
63	0,704	0,693	0,562	0,463	0,624	0,544
64	2,098	0,709	2,508	0,508	1,693	0,596
65	-2,73	0,699	-3,648	0,477	-2,444	0,566
66	0,82	0,703	0,503	0,491	1,058	0,577
67	-0,268	0,707	-0,562	0,504	-0,271	0,589
68	-1,007	0,693	-1,416	0,463	-0,963	0,544
69	-0,366	0,703	-0,597	0,491	-0,501	0,577
7	0,164	0,693	-0,067	0,464	0,401	0,546
70	0,366	0,714	0,044	0,522	0,586	0,61
71	1,713	0,709	1,609	0,507	1,894	0,596
72	0,957	0,693	0,889	0,463	0,836	0,544
73	0,497	0,703	0,257	0,491	0,564	0,577
74	2,329	0,704	2,463	0,497	2,261	0,577
75	0,861	0,697	0,756	0,476	0,807	0,56
76	2,149	0,697	2,155	0,476	2,222	0,56
77	1,239	0,702	1,065	0,491	1,442	0,571
78	1,294	0,705	1,148	0,495	1,401	0,584
79	1,049	0,693	0,891	0,463	1,103	0,544
8	0,629	0,695	0,544	0,471	0,701	0,549

	80	-0,811	0,703	-1,308	0,491	-0,735	0,577
	81	-0,81	0,694	-1,205	0,468	-0,744	0,549
	82	-1,451	0,703	-1,926	0,491	-1,436	0,577
	83	0,635	0,697	0,683	0,476	0,451	0,56
	84	-1,072	0,693	-1,841	0,463	-0,71	0,544
	85	-0,444	0,697	-0,82	0,476	-0,366	0,56
	86	-3,926	0,697	-4,871	0,476	-3,77	0,56
	87	-1,042	0,697	-1,317	0,476	-1,079	0,554
	88	1,694	0,716	1,783	0,526	1,772	0,617
	89	-0,956	0,775	-1,369	0,667	-0,834	0,775
	9	1,511	0,697	1,499	0,476	1,417	0,56
	90	1,632	0,703	1,759	0,491	1,42	0,577
	91	1,401	0,695	1,496	0,471	1,31	0,549
	92	2,255	0,693	2,418	0,463	2,208	0,544
	93	-0,364	0,693	-0,565	0,463	-0,431	0,544
	94	-3,07	0,697	-4,001	0,476	-2,923	0,56
	95	2,065	0,697	2,122	0,476	2,061	0,56
	96	3,018	0,693	3,405	0,463	2,743	0,544
	97	2,067	0,693	2,493	0,463	1,861	0,544
	98	5,94	0,724	6,628	0,546	5,641	0,641
	99	-1,16	0,709	-1,392	0,507	-1,355	0,596
GP137		0,035	0,801	0,282	0,658	-0,125	0,886
GP144		-0,148	0,808	-0,103	0,679	0,109	0,908
GP153		0,106	0,801	0,513	0,658	-0,286	0,886
GP155		-0,866	0,805	-0,979	0,672	-0,602	0,887
GP156		-0,057	0,802	0,157	0,659	-0,21	0,887
GP157		1,26	0,805	2,111	0,671	0,483	0,887
GP205		-0,277	0,823	0,205	0,709	-0,619	0,93
GP232		-0,608	0,801	-0,971	0,658	0,032	0,886
GP246		-0,215	0,801	-0,132	0,658	-0,077	0,886
GP251		0,562	0,802	1,183	0,663	0,471	0,886
GP258		0,596	0,8	1,58	0,656	-0,099	0,886
GP289		0,134	0,807	0,311	0,663	0,475	0,907
GP299		0,057	0,806	0,311	0,677	0,276	0,886
GP324		-0,745	0,814	-0,668	0,685	-0,385	0,93
GP33		-2,173	0,81	-2,407	0,678	-1,35	0,93
GP342		0,554	0,801	1,129	0,657	0,506	0,886
GP363		1,3	0,801	2,309	0,658	0,689	0,886
GP364		-1,191	0,801	-1,142	0,658	-1,126	0,886
GP376		-0,364	0,801	-0,111	0,658	-0,413	0,886
GP387		1,57	0,815	2,297	0,686	1,15	0,93
GP423		0,159	0,814	0,409	0,686	0,396	0,93
GP45		-0,666	0,801	-0,411	0,658	-0,49	0,886
GP457		1,203	0,803	2,008	0,665	0,604	0,887

GP50	-1,795	0,801	-2,127	0,658	-1,142	0,886
GP55	-0,721	0,803	-0,712	0,664	-0,005	0,886
GP554	-0,4	0,806	0,276	0,678	-1,019	0,886
GP56	-1,677	0,858	-1,133	0,791	-1,739	1,012
GP66	-0,774	0,801	-0,642	0,658	-0,338	0,886
GP81	-0,447	0,842	-0,339	0,737	-0,277	0,982
GP86	0,36	0,81	0,827	0,678	0,285	0,93
GR103	1,195	0,801	1,834	0,658	1,118	0,886
GR105	-0,926	0,801	-1,341	0,658	0,238	0,886
GR114	-1,862	0,801	-2,252	0,658	-1,195	0,886
GR126	-0,601	0,801	-0,46	0,658	-0,373	0,886
GR129	0,244	0,801	0,829	0,658	0,048	0,886
GR131	-1,931	0,801	-2,174	0,658	-1,279	0,886
GR133	-0,32	0,801	0,201	0,658	-0,448	0,886
GR135	-0,789	0,806	-0,879	0,678	-0,292	0,886
GR136	-0,158	0,801	0,295	0,658	-0,233	0,886
GR138	-2,174	0,801	-1,774	0,658	-2,333	0,886
GR150	-2,606	0,804	-3,348	0,664	-1,372	0,907
GR160	0,07	0,808	0,63	0,678	-0,133	0,907
GR168	-1,093	0,801	-0,975	0,658	-0,771	0,886
GR221	0,819	0,803	1,605	0,664	0,651	0,886
GR225	-1,39	0,808	-1,716	0,678	-0,505	0,907
GR226	-1,593	0,776	-1,702	0,613	-1,232	0,816
GR232	-0,817	0,801	-0,913	0,658	-0,263	0,886
GR239	-0,438	0,801	-0,288	0,658	-0,205	0,886
GR257	-1,481	0,801	-1,905	0,658	-0,73	0,886
GR263	0,941	0,801	1,548	0,658	0,93	0,886
GR274	-0,076	0,802	0,368	0,659	-0,171	0,887
GR299	-0,902	0,802	-1,063	0,659	-0,836	0,887
GR324	1,464	0,802	2,166	0,659	0,92	0,887
GR332	-0,785	0,811	-1,093	0,68	-0,13	0,931
GR340	0,726	0,801	1,389	0,658	0,145	0,886
GR341	0,993	0,801	1,792	0,658	0,725	0,886
GR347	-0,484	0,802	-0,2	0,659	-0,779	0,887
GR35	-1,44	0,816	-1,416	0,701	-1,358	0,907
GR350	0,508	0,801	0,878	0,658	0,323	0,886
GR354	-1,262	0,801	-1,614	0,658	-0,744	0,886
GR356	-1,233	0,807	-1,134	0,679	-1,28	0,887
GR361	-1,016	0,812	-0,784	0,686	-1,18	0,907
GR362	-0,592	0,801	-0,254	0,658	-0,897	0,886
GR367	0,413	0,81	0,543	0,678	0,549	0,93
GR368	-0,728	0,801	-0,67	0,658	-0,768	0,886
GR371	-0,355	0,801	-0,318	0,658	-0,281	0,886

GR377	-1,993	0,819	-2,194	0,693	-1,914	0,93
GR382	0,901	0,808	1,439	0,679	0,627	0,908
GR383	0,316	0,803	0,68	0,664	0,163	0,886
GR391	-0,579	0,801	-0,671	0,658	-0,265	0,886
GR394	1,574	0,801	1,963	0,658	1,554	0,886
GR397	-1,13	0,801	-1,628	0,658	-0,397	0,886
GR398	0,758	0,801	1,04	0,658	0,743	0,886
GR399	-1,865	0,801	-2,028	0,658	-1,435	0,886
GR404	-2,103	0,816	-2,303	0,701	-1,52	0,93
GR408	-0,043	0,801	0,025	0,658	0,061	0,886
GR411	0,899	0,801	1,492	0,658	0,34	0,886
GR412	-0,182	0,803	-0,072	0,661	0,072	0,889
GR413	0,661	0,803	1,132	0,664	0,406	0,886
GR418	-0,463	0,808	-0,304	0,678	-0,357	0,907
GR42	-2,107	0,801	-2,414	0,658	-1,362	0,886
GR421	-0,148	0,801	0,085	0,658	-0,269	0,886
GR422	0,532	0,802	0,847	0,659	0,406	0,887
GR424	0,402	0,801	0,895	0,658	0,131	0,886
GR426	0,617	0,801	0,98	0,658	0,382	0,886
GR428	1,362	0,801	1,998	0,658	0,946	0,886
GR429	-1,351	0,801	-1,397	0,657	-1,104	0,886
GR431	-1,542	0,8	-1,852	0,656	-0,869	0,886
GR432	0,048	0,8	0,078	0,656	0,469	0,886
GR45	-0,501	0,808	-0,231	0,678	-0,351	0,907
GR455	-0,008	0,801	0,129	0,657	0,393	0,886
GR463	-0,592	0,802	-0,66	0,657	0,091	0,907
GR464	0,112	0,807	0,769	0,677	-0,292	0,907
GR468B	0,042	0,801	0,367	0,657	0,21	0,886
GR473	-0,323	0,801	-0,073	0,657	-0,155	0,886
GR485	-1,293	0,807	-1,39	0,677	-0,856	0,907
GR496	-0,436	0,801	0,012	0,657	-0,687	0,886
GR500	-1,448	0,807	-1,289	0,677	-1,441	0,907
GR502	-0,112	0,801	0,367	0,657	-0,379	0,886
GR506	-0,586	0,801	-0,769	0,657	-0,121	0,886
GR507	1,107	0,802	1,764	0,663	0,952	0,886
GR508	-2,578	0,801	-3,29	0,657	-1,436	0,886
GR515	-2,722	0,806	-3,321	0,677	-1,963	0,886
GR542	0,371	0,801	0,94	0,657	0,362	0,886
GR545	-0,845	0,807	-0,906	0,677	-0,397	0,907
GR548	-0,316	0,801	-0,15	0,657	-0,007	0,886
GR549	0,89	0,814	1,602	0,685	0,749	0,93
GR569	0,747	0,801	1,352	0,657	0,668	0,886
GR570	-1,26	0,805	-0,994	0,677	-1,425	0,886
GR577	-0,334	0,801	-0,243	0,657	0,195	0,886

GR582	-0,746	0,807	-0,804	0,677	-0,279	0,907
GR602	-0,957	0,807	-1,002	0,677	-0,423	0,907
GR62	-0,795	0,801	-1,055	0,658	-0,07	0,886
GR63	-2,075	0,801	-2,472	0,658	-1,24	0,886
GR630	-2,022	0,802	-2,368	0,663	-1,363	0,886
GR633	-0,909	0,803	-1,163	0,67	-0,284	0,886
GR634	-3,491	0,825	-3,97	0,726	-2,516	0,955
GR635	0,371	0,803	1,124	0,67	-0,063	0,886
GR636	-1,851	0,805	-2,149	0,671	-1,312	0,886
GR637	0,81	0,801	1,514	0,658	0,305	0,886
GR638	-2,938	0,81	-3,899	0,687	-1,72	0,908
GR639	0,103	0,801	0,302	0,658	0,32	0,886
GR648	-0,504	0,808	-0,315	0,679	-0,587	0,908
GR652	-0,427	0,814	-0,127	0,701	-0,574	0,907
GR654	1,804	0,801	2,623	0,658	1,465	0,886
GR656	1,52	0,801	2,432	0,658	0,72	0,886
GR657	0,593	0,804	1,222	0,661	0,06	0,911
GR658	0,692	0,801	1,406	0,658	-0,248	0,886
GR659	0,286	0,801	0,641	0,658	-0,006	0,886
GR66	0,336	0,801	0,67	0,658	0,444	0,886
GR661	-1,825	0,805	-2,258	0,671	-1,128	0,886
GR662	0,708	0,803	1,245	0,665	0,366	0,886
GR663	-1,092	0,801	-1,166	0,658	-1,097	0,886
GR664	1,269	0,801	1,975	0,658	0,712	0,886
GR666	0,194	0,805	0,907	0,665	-0,462	0,908
GR669	0,489	0,801	1,125	0,658	-0,387	0,886
GR670	-0,804	0,821	-1,09	0,711	-0,171	0,931
GR672	0,088	0,801	0,66	0,658	-0,727	0,886
GR673	-0,58	0,801	-0,619	0,658	-0,212	0,886
GR674	1,422	0,809	2,302	0,671	0,902	0,907
GR675	-1,29	0,803	-1,684	0,665	-0,518	0,886
GR676	-0,411	0,801	-0,47	0,658	0,054	0,886
GR696	-0,565	0,802	-0,746	0,659	-0,013	0,887
GR697	-1,282	0,801	-1,478	0,658	-1,01	0,886
GR7	-1,341	0,814	-1,87	0,685	-0,264	0,93
GR72	0,093	0,801	0,18	0,658	0,56	0,886
GR738	0,554	0,801	1,042	0,658	0,253	0,886
GR753	-0,095	0,801	0,317	0,658	-0,325	0,886
GR759	-1,297	0,801	-1,664	0,658	-0,586	0,886
GR77	0,833	0,801	1,279	0,658	0,87	0,886
GR770	-2,198	0,81	-2,865	0,693	-1,148	0,886
GR771	-0,253	0,801	0,012	0,658	-0,465	0,886
GR776	0,429	0,801	0,939	0,658	-0,02	0,886

GR786	0,247	0,801	0,515	0,658	0,239	0,886
GR793	1,464	0,812	2,204	0,686	0,996	0,93
GR80	0,205	0,802	0,629	0,659	0,252	0,887
GR82	-0,163	0,801	0,324	0,658	-0,172	0,886
GT1	-3,128	0,647	-2,776	0,291	-3,928	0,38
PB235	-1,753	0,671	-2,208	0,361	-1,01	0,491
RRIM701	-1,694	0,67	-2,487	0,353	-0,685	0,487

Supplementary Table 3. Expected genetic gain (EGG) using the classical breeding method (CBM) or genomic prediction with the single-environment, main genotypic effect model (SM); with the multienvironment, genotypic effect model (MM); with the multienvironment, single variance G×E model (MDs); and with the multienvironment, environment-specific variance G×E model (MDe) using the GB and GK matrix in the two evaluated environments, LW and WW.

Model	matrix	env	PA	EGG
CBM	-	-	-	0,08
	-	LW	-	0,07
	-	WW	-	0,07
MDe	GK	LW	0,83	0,39
	GB	LW	0,82	0,38
	GK	WW	0,87	0,41
	GB	WW	0,86	0,41
MDs	GK	LW	0,84	0,39
	GB	LW	0,83	0,39
	GK	WW	0,87	0,41
	GB	WW	0,86	0,41
MM	GK	LW	0,84	0,39
	GB	LW	0,83	0,39
	GK	WW	0,86	0,41
	GB	WW	0,86	0,4
SM	GK	LW	0,19	0,09
	GB	LW	0,19	0,09
	GK	WW	0,28	0,13
	GB	WW	0,28	0,13

Supplementary Figures

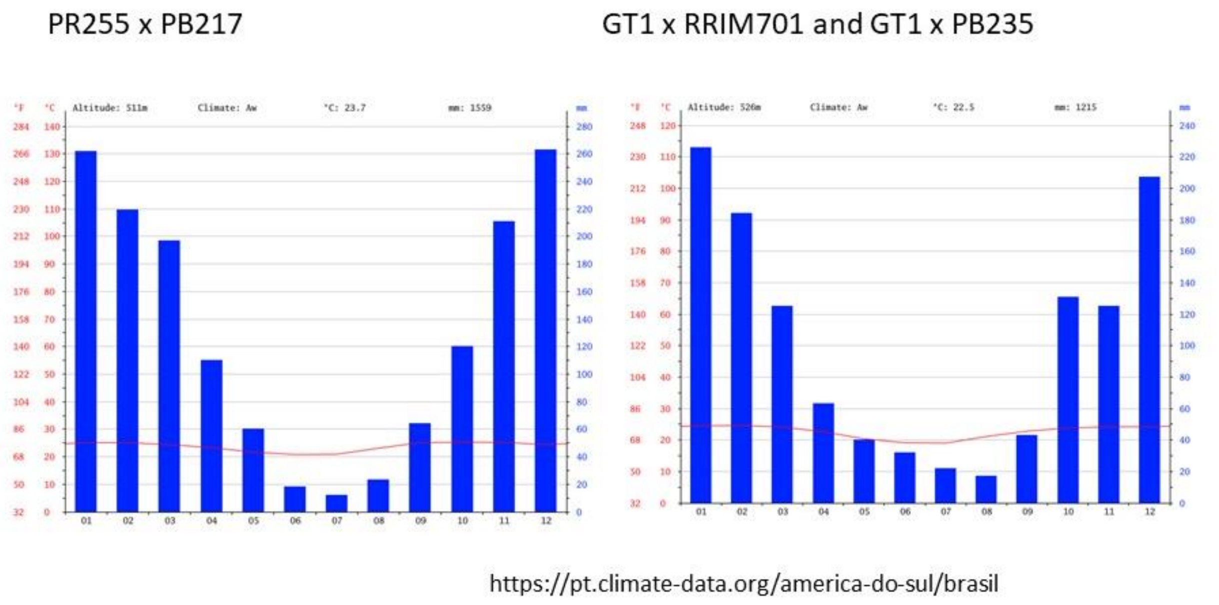

Supplementary Figure 1. Climate data of Votuporanga (GT1 x RRIM 701 and GT1 x PB235) and Itiquira (PR255 x PB 217) with average temperature and precipitation values throughout the months of the year.

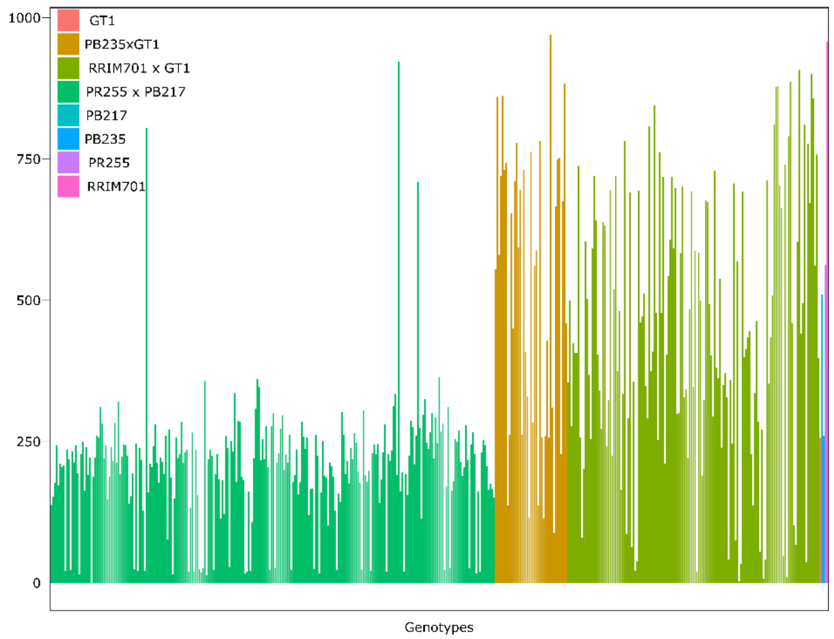

Supplementary Figure 2. Mean sample read depths for SNPs identified in 435 rubber tree genotypes using the GBS approach.

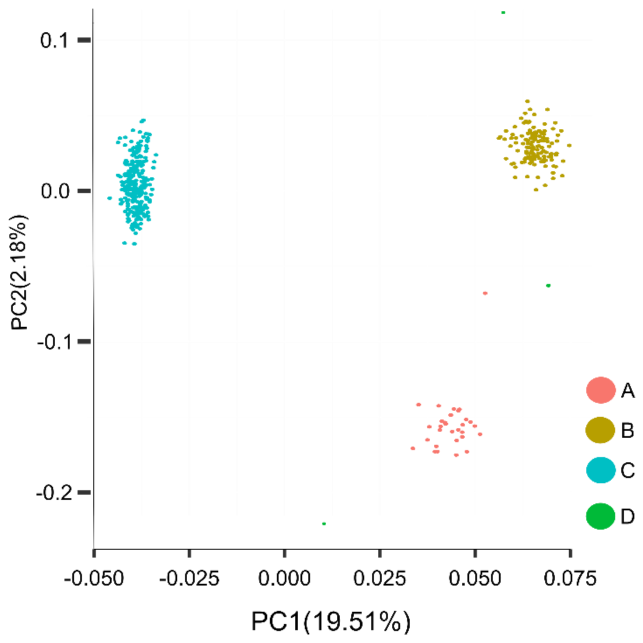

Supplementary Figure 3 Population structure analysis of 411 rubber tree genotypes with the first two principal components (PCs).

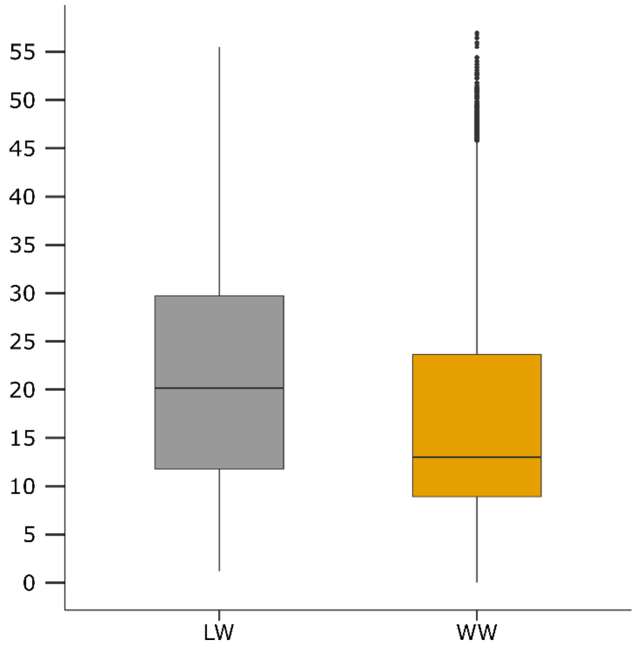

Supplementary Figure 4. Box plot of stem circumference (SC) in the two environments: WW (well-watered) and LW (low-water).
